# Orthodontia Notes

**Published:** 1906-07-15

**Authors:** W. J. Brady


### ORTHODONTIA NOTES.

BY W. J. BRADY,

(In "Western Dental Journal.)

Orthodontia.—Orthodontia treatment is neither a fad, fancy nor fallacy, but one of the great necessities of life, just as much needed as eye glasses or medicine.

Time for Correction.—All things considered, the best time for regulating is between the ages of eight and thirteen years, the preference always being for early operation.

The earlier notice is taken of irregularity the better, as its steady increase is then noted and treatment may be induced before it is too late.

---

Bending the Arch.—The expansion arch often produces too great outward movement of the molar teeth, due to too much spring upon the molars, and too little space between the bicuspid teeth and the arch. Expand the anterior portion of the dental arch first, ligating the bicuspids (and cuspids if necessary), very tightly to the arch, and making little effort to expand in the molar region until toward the last of the operation. The fact that the expansion arch sometimes displaces the molars is due largely to failure to observe the foregoing details.

---

Removing Bands.—Cemented bands may be easily and quickly removed from teeth with a thin screw driver shaped instrument, made from an old excavator or similar instrument. Insert the blade of this underneath the band, then partially rotate first one way and then the other, and start the band to tear in too. Follow up this break turning the instrument to right and left, and it is quickly torn entirely across. This is much easier on the patient as well as better for the tooth than to cut in two with chisel, fissure bur, or carborundum wheel.

---

An Unquestioned Duty.—The general practitioner first meets cases of mal-occlusion, and it is his solumn duty to call attention to the same and the benefits and possibilities of treatment. It is little short of inhuman to allow children in these days to grow up deformed and make no effort to relieve them. If a specialist is within reach cases should be sent to him that he may at least fully inform parents what may be done, and in nine cases out of ten they will be grateful beyond words that their attention is called to such matters.

Gold Plating.—So far as experience has shown up to the present time, it is impossible to gold plate any base metal so that it will not tarnish in a short time when placed in the mouth. Every expedient known in the plater's art has been tried, even to the extent of plating four or five deposits of nickel and copper, but the effect seems the same in all—the plating is lost in a short time. The best results are obtained by triple plating, *i. e.*, three coats, one after the other, with a thorough burnishing between each coat, using cold solutions and a current of very low voltage. This makes a hard and fine-grained deposit, not of such brilliant color as by other methods, but the most durable that has been produced. Even this, however, tarnishes through in a few weeks in most mouths.

---

Tempering Wire.—Wire of german silver or other alloys is given its temper altogether by the method in which it is drawn down, and is not tempered by heating and cooling, as with steel. The reduction of very little each time for a large number of drawings will make the wire soft and very tough; the reduction of a considerable amount each time for a few drawings will make the wire very hard and brittle; the reduction of a medium amount for a few drawings gives a spring temper. Each metal must be handled in a different way, and the production of spring temper is by no means easy. Spring temper is lost by over-heating or over-bending, points which should be always remembered in handling appliances.

